# Reduction of Death Rate Due to Acute Myocardial Infarction in Subjects with Cancers through Systemic Restoration of Impaired Nitric Oxide

**DOI:** 10.1371/journal.pone.0088639

**Published:** 2014-02-18

**Authors:** Rajeshwary Ghosh, Udayan Ray, Pradipta Jana, Rabindra Bhattacharya, Debipriya Banerjee, Asru Sinha

**Affiliations:** 1 Sinha Institute of Medical Science and Technology, Kolkata, India; 2 Royal Hobart Hospital, University of Tasmania, Australia; 3 Kalyani Gandhi Memorial Hospital, West Bengal, India; Goethe University, Germany

## Abstract

**Introduction:**

Excessive aggregation of platelets at the site of plaque rupture on the coronary artery led to the formation of thrombus which is reported to precipitate acute myocardial infarction (AMI). Nitric oxide (NO) has been reported to inhibit platelet aggregation and induce thrombolysis through the *in situ* formation of plasmin. As the plasma NO level in AMI patients from two different ethnic groups was reduced to 0 µM (median) compared to 4.0 µM (median) in normal controls, the effect of restoration of the NO level to normal ranges on the rate of death due to AMI was determined.

**Methods and Results:**

The restoration of plasma NO level was achieved by a sticking small cotton pad (10×25 mm) containing 0.28 mmol sodium nitroprusside (SNP) in 0.9% NaCl to the abdominal skin of the participants using non-toxic adhesive tape which was reported to normalize the plasma NO level. The participants (8,283) were volunteers in an independent study who had different kinds of cancers and did not wish to use any conventional therapy for their condition but opted to receive SNP “pad” for their condition for 3 years.

The use of SNP “pad” which normalized (≈4.0 µM) the plasma NO level that in consequence reduced the death rate due to AMI, among the participants, was found to be significantly reduced compared to the death due to AMI in normal population.

**Conclusion:**

Our data suggested that the use of SNP “pad” significantly reduced the death due to AMI.

**Trial Registration:**

www.ctri.nic.in 004236

## Introduction

The aggregation of platelets by aggregating agents like ADP, *l*-epinephrine, collagen or thrombin is a critically important physiologic event in the life saving blood coagulation process [1]. In contrast, the formation of thrombus (microaggregate of platelet embedded in fibrin mass) due to excessive platelet aggregation, particularly by ADP [2], on the major or medium sized coronary artery, at the site of atherosclerotic plaque rupture or fissuring has been reported to result in acute coronary syndromes leading to unstable angina or acute myocardial infarction [3]. In acute cases, the thrombus induced blockade of the normal blood circulation would not only impede the availability of the oxygenated blood, but would also interrupt the supply of nutrients, minerals and water that are essential for the normal activities of the cardiac muscles leading to acute ischemic heart disease (AIHD) including acute myocardial infarction (AMI). And, as such, the formation of the thrombus on the coronary arterial wall plays a critically important role in the pathogenicity of coronary artery disease (CAD). On the other hand the dissolution of the formed thrombus might help to resolve the condition through the restoration of normal blood circulation in the heart muscles. Various thrombolytic agents are used to achieve the resolution of the formed thrombus [4]–[8].

Excessive aggregation of platelets is affected by various platelet aggregating agents leading to thrombus formation. However, the excessive platelet aggregation is counteracted by the effect of several humoral inhibitors of platelet aggregation including prostacyclin [9], interferon-α [10], and insulin [11]. While prostacylin (PGI_2_) is reported to inhibit platelet aggregation through the cellular increase of cyclic AMP level [12], insulin and interferon-α are reported to inhibit platelet aggregation by increasing the cellular synthesis of nitric oxide (NO) [13], [10]. Nitric oxide has been reported to inhibit platelet aggregation not only through the cellular increase of both cyclic AMP and GMP level [14]–[16], NO can also inhibit platelet aggregation through the direct activation of plasminogen to plasmin [10] which in turn inhibited the platelet aggregation through the dissolution of inter platelets' fibrinogen bridges even in the absence of both cyclic AMP and cyclic GMP syntheses in platelets [17], [18].

The inhibition of platelet aggregation by acetyl salicylic acid (aspirin) is not only reported to inhibit platelet aggregation through the inhibition of platelet cyclooxygenase [19], but the compound is also reported to induce NO synthesis in platelets and in other cells [20]. Nitric oxide thus produced is reported to dissolute inter platelets' fibrinogen bonds by the formation of plasmin from plasminogen [10], [20]. Extensive studies have demonstrated that the use of aspirin significantly reduced the occurrence of CAD [21], [22], and it has been proposed that the efficacy of aspirin to reduce the occurrence of CAD was related not only to the ability of the compound to inhibit platelet cyclooxygenase [23], [24], but also to the thrombolytic effect of the compound due to NO synthesis leading to plasmin formation [25]. In this context it could be mentioned that either the direct injection of NO in 0.9% NaCl or the injection of insulin that stimulated systemic NO synthesis in mice were found to effectively protect the animal from death due to coronary thrombosis induced by the injection of ADP in the circulation even in the absence of atherosclerotic plaque rupture in the “normal” animals [26].

In a preliminary study, to determine the possible role of systemic NO in CAD, if any, when the plasma NO level was determined in patients suffering from AMI, it was found that the plasma NO level in these patients (n = 125; M = 101, F = 24) was severely reduced (0 nmol/ml; (median). The results of the preliminary study and the role of NO as an antiplatelet and a thrombolytic agent as reported above, prompted us to assess the possibility of preventing AMI, through the systemic increase of the plasma NO level by using sodium nitroprusside (SNP) “pad” as described before in animal model as well as in humans [27], [28]. As it was neither practicable nor feasible to use SNP “pad” indefinitely for the systemic increase of NO level in normal volunteers, to determine the effect of increased plasma NO level to reduce the occurrence of AMI, the effect of the increase of plasma NO levels in the prevention of AMI in subjects with cancers was carried out for two different reasons: 1) cancer patients are reported to be at a higher risk of death due to AMI compared to normal populace [29]–[33], and 2) The study (participation of the cancer patients) was not particularly initiated to determine the effect of increased systemic NO level by using SNP “pad” (please see below) on the incidences of AMI in the cancer patients. These cancer patients participated in an independent study and their inclusion was related only to a serendipitous observation where the subjects who received SNP “pad” had remarkably lower death rate due to AMI compared to the normal population. It should be noted here that the outcomes evaluated in this paper were not part of the original clinical trial and this was instead a secondary analysis of the results obtained before [28]. These subjects with different kinds of cancers, who participated in our study had opted at their own wish not to receive any chemotherapy, radiation or surgery for the condition but wished to receive only dermal “antineoplastin” therapy which is actually an SNP “pad” [28]. It should be mentioned here that these volunteers were already receiving dermal SNP “pad” for the past 3 years for their neoplastic conditions when the death rate among these patients was compared to normal volunteers and analyzed only for statistical purpose. The effects of the systemic increase of plasma NO level in the prevention of AMI in the animal model as well as in humans with cancer who were reported to be predisposed to the increased occurrence of AMI compared to that in normal population [29]–[33] are presented herein.

## Materials and Methods

The protocol for this trial and supporting checklist are available as supporting information; see Checklist S1 and Protocol S1.

### Ethics Statement

This study requested the participation of both normal volunteers and age and gender- matched persons with different kind of cancers. These cancer patients at the time of presentation were receiving only “antineoplastin” dermal pad, a novel anticancer therapy as described before [28]. Briefly, “antineoplastin” pad when applied to the skin of the recipient has been reported to increase the plasma NO level, through the “feedback” activation of insulin activated nitric oxide synthase (IANOS) in the formed elements of the blood as well as in the endothelial cells of the vessels [34]. These patients by their own wish did not opt to receive any other therapy simultaneously. The appropriate approval for the use of the dermal “antineoplastin” (SNP “pad”) by these volunteers with different forms of cancers was obtained from the Institutional Review Board for Human Research, Sinha Institute of Medical Science and Technology, Kolkata, India, and from the Calcutta High Court of Law only after legal counselling was made available to each of these volunteers in the presence of their family members as reported before (Protocol S1) [28]. Each participant was given free access to counsel a lawyer and a family member to obtain permission. No volunteers with cancer were allowed to participate in the study without written consent (judicial affidavit) as signed by a magistrate and when the participants were selected, they were asked to sign an informed consent form in the presence of a witness. All the participants were thoroughly advised on both the benefits and risks in the presence of a legal counsellor with the understanding that the participant may quit the research activity without prior permission from the investigators. No economic compensation was asked either by the participants or by the investigators in any phase of the work. The verdict for pursuing the research activity on the cancers as described above using “antineoplastin” was approved by the highest court of law of the state i.e., The Calcutta High Court. Also please note that extensive experiments were performed in three types of animals as asked by the Calcutta High Court. The results of the investigation and the approval by the High Court are available in public domain for inspection by any concerned person.

The plasma samples from AMI which were from Australia were also used in the study and necessary permission was obtained from the Human Research Ethics, University of Tasmania, Australia.

This study also used healthy white Swiss Albino mice after being examined by a certified veterinarian. A standard pellet diet and sterile water were given *ad libitum*. Appropriate permission was obtained from the Institutional Review Board for Animal Care, Sinha Institute of Medical Science and Technology, Kolkata, India. Care was taken to ensure that no animals were unnecessarily harmed or were subjected to pain during the study and the studies were performed only in the presence of a member belonging to the Animal Right Group. After the termination of the study, the animals were sacrificed by euthanasia using a CO_2_ chamber.

### Selection of patients with different cancers

**Figure 1:**
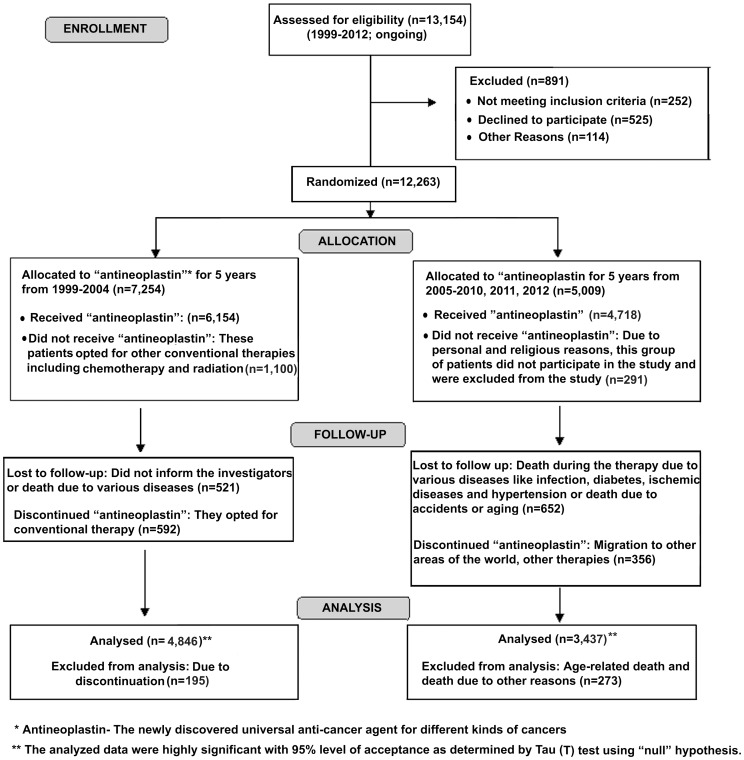
Flowchart representing the participation of the subjects with different cancers who received “antineoplastin” treatment. Patients with different cancers were selected for the study and “antineoplastin” pad consisting of 0.2 ml of the solution (0.28 mmol of SNP) soaked in a band-aid was applied dermally on the lower abdominal hair-free area as described before [28]. Please note that the whole process of delivering the “pad” containing “antineoplastin” takes less than 5 min. People from different parts of the world belonging to multi-ethnic and multi-cultural backgrounds have participated in the study including India, Poland, Papua New Guinea, Sri Lanka and Bangladesh. They had used this “antineoplastin” pad for at least a year with a maximum duration of 5 years.

The details of the selection of cancer patients and the use of “antineoplastin” pad in the selected patients are shown in Fig. 1. The study was carried out in Sinha Institute of Medical Science and Technology, Kolkata, India, from the year 1999–2012 and is still being continued as per the order of the Calcutta High Court of law (Fig. 1). The “antineoplastin” pad was delivered to the patients by the attending technicians in the presence of medical personnel with M.D. degree. A double-blind method of investigating the study was performed where neither the investigator nor the medical personnel had any knowledge about the type of cancer. Only after a month, when the patients showed comparative improvement related to the complete blood picture, pain and other cancer related problems as confirmed by fine needle aspiration cytology, ultrasonography, hematological test and liver function test, the data were tabulated by an independent tabulator who had no knowledge about the cancer patients or whether or not “antineoplastin” pad was received by these patients [28].

### Selection of normal volunteers

Age and gender matched normal volunteers also participated in the study. These persons at the time of participation in the study were not suffering from hypertension nor had the history of diabetes mellitus. None of the normal volunteers were hospitalized for any condition within the last 6 months and none had life-threatening infection. Appropriate informed consent form was signed by these volunteers.

### Selection of patients with acute myocardial infarction (AMI)

To minimise the influence of ethnic background and geographical location on the plasma NO level in AMI patients, subjects from both Calcutta in India, and from Hobart, Tasmania in Australia were asked to participate in the study.

#### Inclusion criteria for all AMI

In Calcutta, all patients were admitted to the intensive care unit of Calcutta Medical College and Hospital, Calcutta, India, (n = 55; M = 45; F = 10; median age 59 yrs). In Australia, patients were admitted to Royal Hobart Hospital, Tasmania, Australia (n = 70; M = 56; F = 14; median age 54 yrs). All the participants had chest pain characteristics of myocardial ischemia for 30 min or more. The electrocardiogram showed typical ST elevation of at least two leads in ECG indicating a single myocardium region. The confirmation of the condition was determined by the elevated creatine kinase and creatine kinase-MB isoenzymes in the blood samples. The sampling of the blood was made within 6 h of the onset of the anginal attack before the initiation of any therapy for the condition.

#### Exclusion criteria

Patients with history of diabetes mellitus, cerebrovascular accident or showing the presence of bundle branch block or left ventricular hypertrophy in the ECG that did not allow the diagnosis of ischemia/infarction or had life-threatening infection were excluded from the study.

However, although the participating patients at presentation had no history of the diseased conditions as mentioned above, some of the patients during the course of the study did develop the cardiovascular risk factors viz., hypertension and diabetes mellitus. These patients were appropriately treated by using anti-hypertensive and hypoglycemic drugs as considered by the attending physician independent of the study (Table 1).

**Table 1:** Characterization of the acute myocardial infarction in patients selected for the study.

ACUTE MYOCARDIAL INFARCTION (INDIAN PATIENTS) (n = 55; M = 45; F = 10)					ACUTE MYOCARDIAL INFARCTION (AUSTRALIAN PATIENTS) (n = 70; M = 56; F = 14)				
No. of patients	Gender and Age	Co-morbidities and cardiovascular risk factors	NO (nmol/h)	Medications	No. of patients	Gender and Age	Co-morbidities and cardiovascular risk factors	NO(nmol/h)	Medications
1	M (65)	-	0	-	1	M(70)	HYP; Smoker	0.001	b
2	M(70)	Smoker; DM	0.002	a	2	M(47)	-	0.021	-
3	M(47)	HYP; Smoker	0	b	3	M(54)	-	0.051	-
4	M(55)	-	0.12	-	4	M(57)	Smoker	0	-
5	M(75)	HYP	0	b	5	M(55)	HYP; DM; Smoker	0	a and b
6	F(70)	DM	0	a	6	M (65)	-	0.021	-
7	M(76)	DM; Smoker	0	a	7	M(59)	-	0.054	-
8	M(59)	HYP	0	b	8	M(65)	-	0.21	-
9	M(65)	Smoker	0.25	-	9	M(68)	HYP; Smoker	0	b
10	M(68)	-	0.55	-	10	F(65)	HYP;DM	0	a and b
11	F(65)	HYP	0	b	11	M(68)	HYP	0	-
12	F(70)	DM	0	a	12	M(58)	-	0	-
13	M(68)	HYP; Smoker	0	b	13	F(71)	HYP	0	b
14	M(72)	-	0.5	-	14	F(75)	HYP;DM	0	a and b
15	M(71)	DM; Smoker	0.7	a	15	M(56)	HYP; Smoker	0	b
16	F(65)	DM	0	a	16	M(50)	HYP	0.002	b
17	M(56)	HYP; Smoker	0.3	-	17	F(52)	-	0	-
18	M(50)	HYP; DM	0.012	a and b	18	M(38)	HYP;DM	0	a and b
19	M(38)	-	0	-	19	M(65)	HYP	0	b
20	M(66)	HYP; Smoker	0	b	20	M(64)	DM	0	a
21	M(80)	-	0	-	21	M(70)	DM; Smoker	0	a
22	M(68)	-	0	-	22	M(66)	-	0.321	-
23	M(39)	-	0.32	-	23	F(57)	-	0.351	-
24	M(44)	Smoker	0.021	-	24	M(37)	-	0.002	-
25	F(60)	HYP	0.025	b	25	M(43)	HYP	0.005	b
26	M(55)	HYP; Smoker	0	b	26	M(53)	-	0.004	-
27	M(50)	HYP; Smoker	0	b	27	M(50)	DM; Smoker	0	a
28	M(49)	-	0.251	-	28	M(59)	Smoker	0	-
29	M(59)	HYP	0	b	29	M(60)	DM; Smoker	0	a
30	M(50)	HYP; DM	0	a and b	30	M(60)	HYP; Smoker	0	b
31	M(60)	-	0	^-^	31	F(61)	-	0.21	b
32	M(51)	Smoker	0	^-^	32	M(64)	-	0.123	-
33	M(60)	DM; Smoker	0.023	a	33	F(51)	-	0.004	-
34	M(61)	Smoker	0.021	-	34	M(60)	HYP; Smoker	0	-
35	M(60)	HYP; Smoker	0.002	b	35	F(51)	-	0	-
36	F(51)	-	0.015	-	36	M(69)	Smoker	0	-
37	M(84)	Smoker	0.254	-	37	M(69)	HYP; Smoker	0	b
38	M(69)	-	0	-	38	F(60)	-	0.321	-
39	M(74)	-	0	-	39	M(59)	HYP	0.21	b
40	M(70)	-	0	-	40	M(84)	-	0	-
41	M(51)	Smoker	0.32	-	41	M(61)	DM; Smoker	0	a
42	F(73)	HYP; DM	0.25	a and b	42	F(76)	-	0	-
43	M(51)	HYP	0	b	43	M(51)	HYP	0.25	-
44	M(57)	HYP	0	b	44	M(67)	HYP; Smoker	0.321	b
45	M(59)	-	0.35	-	45	M(38)	Smoker	0	-
46	F(49)	-	0.002	-	46	M(64)	HYP; Smoker	0	b
47	F(61)	HYP	0.002	b	47	M(70)	HYP; DM; Smoker	0	a and b
48	M(70)	HYP; Smoker	0.045	b	48	M(62)	HYP; DM; Smoker	0.354	a and b
49	M(62)	Smoker	0.022	-	49	M(82)	HYP; DM; Smoker	0	a and b
50	M(52)	Smoker	0.005	-	50	M(39)	Smoker	0	-
51	M(39)	Smoker	0	-	51	M(73)	HYP; Smoker	0.360	b
52	M(63)	HYP; Smoker	0	b	52	F(68)	HYP; DM	0.202	aand^b^
53	F(68)	HYP	0	b	53	F(61)	DM	0	a
54	M(58)	-	0	-	54	M(58)	DM; Smoker	0	a
55	M(64)	-	0.021	-	55	M(74)	HYP; Smoker	0	b
					56	M(51)	HYP; DM; Smoker	0	a and b
					57	M(67)	DM; Smoker	0	a
					58	M(73)	DM; Smoker	0	a
					59	M(71)	-	0	-
					60	F(65)	HYP	0	b
					61	M(72)	HYP	0	b
					62	M(69)	-	0.32	b
					63	F(50)	HYP	0.451	a
					64	M(54)	Smoker	0.214	-
					65	M(82)	HYP; Smoker	0	a
					66	M(52)	DM; Smoker	0	-
					67	M(64)	Smoker	0	a
					68	M(49)	-	0.254	-
					69	M(65)	DM; Smoker	0.125	-
					70	M(73)	Smoker	0	-

The use of medications for the control of major atherosclerotic risk factors i.e., hypertension and type 2 diabetes mellitus was studied independently under the discretion of the care provider. These conditions were controlled by using various medications as indicated in the Table.

a For those patients suffering from diabetes mellitus, oral hypoglycemic drugs such as sulfonylurea, alpha-glucosidase inhibitor, thiazolidinediones, biguanides, were administered under the supervision of a medical practitioner as found appropriate

b Anti-Hypertensive drugs such as hydroquinone, ACE-inhibitors, Alpha-Adrenoceptor Blockers; (*Prazosin, Doxazosin, Terazosin,Phenoxybenzamine*); Angiotensin II Receptor Blockers, Potassium Channel Opener; Ca^+^ion channel blockers were used in case of the patients who were suffering from hypertension. These patients were mostly suffering from essential hypertension for which neither any specific diagnosis nor any therapy is currently available.

The table represents the various AMI patients who participated in the study. Co-morbidities like hypertension, diabetes mellitus and smoking have been listed in the table. Plasma nitric oxide (NO) values have also been shown for each individual.

HYP =  Hypertension; DM =  Diabetes Mellitus; M =  Male; F =  Female; Numbers within parentheses represent the age of the respective persons.

### Chemicals

ADP was purchased from Sigma Chem. Co. Sodium nitroprusside was a product of Abraxis Pharm. All other chemicals used in the study were of analytical grade.

### Development of coronary thrombosis in mice

Swiss white albino mice were used to develop coronary thrombosis by injecting ADP in the tail vein as described before [26]. These animals were inbred in our animal facility from birth up to the use of 2 months. They were fed standard laboratory chow, and sterilized water was given *ad libitum*. The animals were grown in 12 h cycles of light and darkness. Before use these mice were checked by a licensed veterinarian to determine that they were free of diseases.

### Stimulation of plasma NO levels in animal and human volunteers in vivo

The increase of plasma NO level was achieved by sticking a sterile cotton pad (25 mm X 10 mm) soaked with 1.6 M sodium nitroprusside dehydrate (SNP) in 0.9% NaCl containing 0.28 mmol of SNP to the hair free area of the abdomen by using non toxic adhesive tape as described before [27], [28]. When needed the SNP “pad” was changed every 24 h. The details of the mechanism of increased synthesis of NO by the dermal application of the SNP “pad” have been reported before [28].

### Determination of plasma NO level

The plasma NO level was determined by methemoglobin assay as described before [20]. Typically, the presence of NO in the sample converts oxyhemoglobin to methemoglobin with characteristic changes of the absorption maxima at 575 nm and 600 nm. The formation of methemoglobin was determined by measuring the optical density at these absorption maxima under N_2_ by using a Beckman spectrophotometer (Model DU) as described before [20]. The quantitation of NO was validated by an independent chemiluminescence method [35].

### Collection of blood samples

Peripheral blood was collected by venipuncture by using 19 gauge siliconized needle from the participants in plastic vials and anti coagulated by mixing 9 vol of blood with 1vol of 0.013 M sodium citrate as the anticoagulant, and mixed by gentle inversion. Cell free plasma (CFP) was prepared by centrifuging the blood samples at 10,000 g for 30 min at 0°C.

### Determination of the plasma dermcidin isoform 2 levels

Dermcidin isoform 2, an 11 kDa protein that is reported to appear in the plasma of the patients suffering from AMI, was determined to be a potent inhibitor of all known forms of nitric oxide synthase [36]. Dermcidin was measured by enzyme linked immunosorbent assay (ELISA) by using electrophoretically pure dermcidin isoform 2 as the antigen as reported before [36].

### Preparation of Goat Carotid Artery Endothelial Cell Homogenate

Endothelial cells from the carotid artery of the freshly slaughtered goat was prepared in Tyrod's buffer pH 7.4 as described before [37]. The disrupted cell mass was centrifuged at 60,000 g for 30 min at 0°C. The supernatant was used as the source of endothelial nitric oxide synthase (eNOS).

### Lineweaver-Burk Plot of Insulin-Activated eNOS in the Endothelial Cell Homogenate in the Presence or Absence of Dermcidin

A total volume of 1.0 mL of the reaction mixture contained different concentrations of *l*-arginine in the presence of 2 mM CaCl_2_ with 100 µunits of insulin/mL and in the presence or absence of 0.1 µM dermcidin in Tyrod's buffer, pH 7.4. The product NO was determined after 20 min of incubation at 37°C under N_2_, (during the steady state of formation of NO (1–30 min) as described before [38].

### Statistical analysis

The significance of the prevention of death due to ADP induced coronary thrombosis by using SNP “pad” in the animal model was determined by Student's “t” test. The significance of the survival rate in these animals was determined by Kaplan-Meir survival analysis. In case of human volunteers, the significance of the reduction of plasma NO levels in both the Indian and Australian AMI patients was analyzed by a regression model. The significance of the prevention of death due to AMI in the persons with different kinds of cancers who participated and received SNP “pad” in the study was analyzed by the Z test. The results were considered significant when the null hypothesis was rejected with the calculated z-value greater than the critical two-tailed z-value and P<alpha 0.5. The statistical analyses were performed by Graphpad Prism software.

## Results

### The plasma levels of NO and plasma dermcidin level in AMI patients in Australia and in India and in the gender and aged matched normal volunteers

As described above, to minimize the influences of ethnic and geographical locations of the participants in the plasma NO levels in AMI, the plasma NO level in this condition was determined both in Calcutta, India and in Hobart, Australia.

**Figure 2:**
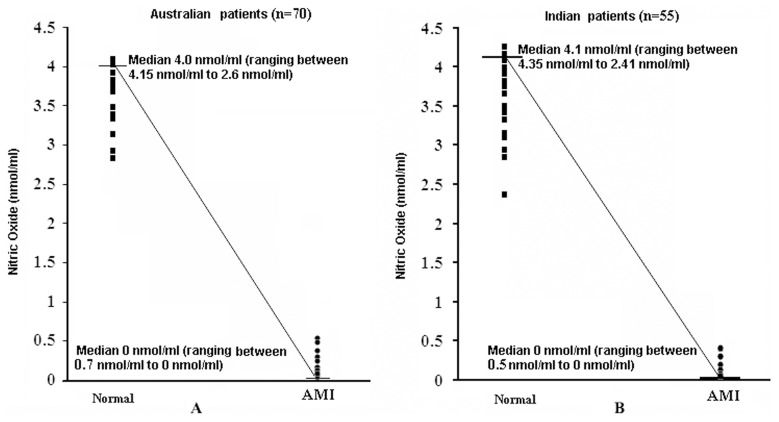
Nitric oxide levels in AMI patients in South Asia and in Southern Australia. The NO level was determined by methemoglobin method as described in the Materials and Methods section in both the cases. Panel A represents the median values of plasma NO level in AMI patients and in the equal number of age and gender matched normal volunteers in Australia. Panel B shows the median values of plasma NO level in AMI patients and in the age and gender matched normal volunteers in India.

It was found that the plasma NO level in the Australian patients (n = 70) was 0 nmol/ml (median) (ranging from 0.07 nmol/ml to 0 nmol/ml) (Fig. 2A) and the plasma NO level in the Indian counterparts (n = 55) also had a median value of 0 nmol/ml (ranging between 0.05 nmol/ml to 0 nmol/ml) (Fig. 2B). These results suggested that neither the ethnic background nor the geographical location of the AMI patients influenced the plasma NO levels in AMI and the plasma NO levels were essentially similar in both the cases. It was further noted that the plasma NO levels both in the males and females were similar in ranges.

**Figure 3:**
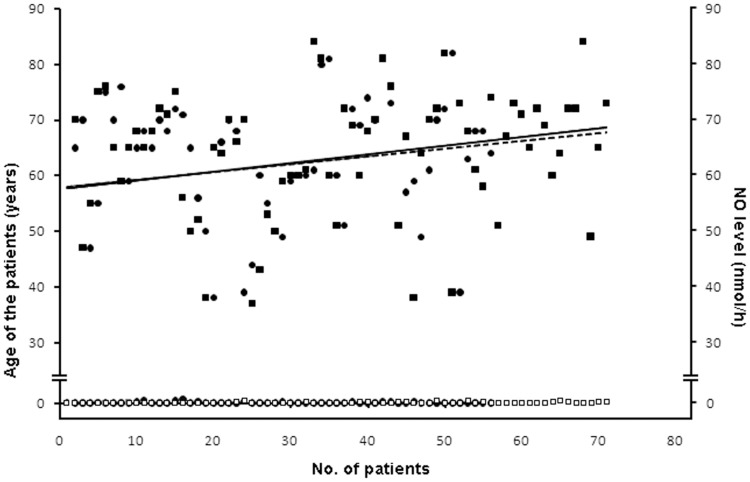
The age group-plasma NO level adjusted regression model in both Indian and Australian AMI patients. Regression model of the age related to the plasma NO level in both the Indian and Australian AMI patients was plotted. The age and the corresponding plasma NO level of the participants could be determined from the plot. Solid line represents the regression curve of the age group of the Indian AMI patients. Dotted line indicates the regression curve of the age group of the Australian AMI patients. Solid circles (•) indicate variations in the age group of Indian AMI patients. Solid squares (▪) indicate variations in the age group of the Australian AMI patients. The hollow circles (○) and the hollow squares (□) represent the plasma NO level in Indian and Australian patients respectively.

Regression curve of the plasma NO level and the age group demonstrated that the occurrence of AMI was prevalent in similar ranges of age group and the plasma NO level was found to hover around 0 nmol/h in both the groups of patient (Fig. 3). It can be concluded that both in Indian and Australian patients the age group and NO level are significant with r^2^ = 0.8021.

### The plasma dermcidin levels in AMI subjects and in age and gender matched normal volunteers

While the plasma dermcidin level in normal volunteers was found to be 9.89±3.9 nM, it increased to 115.5±9.9 nM in AMI patients (p<0.0001; n = 125). The mechanism of dermcidin induced inhibition of NO was studied in a separate experiment where dermcidin was found to inhibit insulin activated nitric oxide synthase in the goat endothelial cells, thereby leading to the inhibition of NO from *l*-arginine. Lineweaver-Burk Plot suggested that dermcidin at the range of 0.1 µM increased the Km from 9.03 µM to 25.45 µM arginine with concomitant decrease of the Vmax from 6.5 nmol NO/h/mg to 4.0 nmol NO/h/mg.

### The protection of mice from death due to ADP induced coronary thrombosis induced by the increase of plasma NO level using SNP “pad”

Coronary thrombosis was induced in mice as an animal model by injecting ADP in the tail vein as described in the Materials and Methods. The injection of ADP in the tail vein of the mice caused death in these animals within 20 mins with the development of thrombus in the coronary artery as described before [26].

To determine the effect of the systemic increase of NO level on the ADP induced death due to the formation of coronary thrombosis, same concentration of SNP “pad” as in the case of human volunteers (0.28 mmol) was applied to the hair freed area of abdomen in the mice as described. The plasma NO level was determined at different times after the application of the “pad”. It was found that the plasma NO level which was 2.1±0.2 µM at zero hour (i.e., before the application of the “pad”) increased to 4.1±0.3 µM after 24 h of the dermal application of the SNP “pad” (p<0.0001; n = 10). At 24 h after the application of the “pad”, the animals were challenged by injecting 1.5 µmol ADP/g body weight in the tail vein. It was found that the animals were protected from death induced by the injection of ADP (at least for 2 days). In contrast, the injection of ADP resulted in the death of the mice within 20 mins where the animals did not receive any SNP “pad” with the plasma NO level at 2.0±0.2 µM (p<0.0001; n = 10) that was similar to that in the control animal (untreated with the “pad”).

The change of the nitroprusside “pad” every 24 h that restored the plasma NO level to 4.2±0.2 µM was found to protect the animals from the death indefinitely due to the repeated thrombogenic assault induced by the repeated injection of ADP every 24 h at least for 7 days.

**Figure 4:**
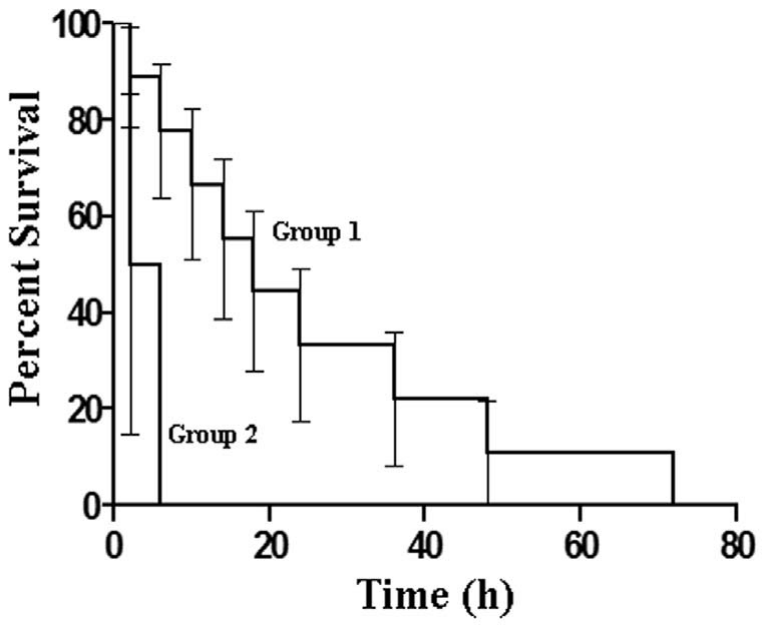
Kaplan-Meier model of the survival rate of the animals with ADP induced coronary thrombosis in the presence and absence of the application of dermal SNP “pad”. To determine the survival rate in the animals from coronary thrombosis by the dermal application of SNP “pad” induced by ADP, Kaplan-Meier survival curve was plotted. Group 1 represents the animals that received the dermal application of SNP “pad”. The curve indicates the survival period which shows survival of the animals up to 72 hours after the repeated injection of ADP. Group 2 represents the control group without SNP “pad” treatment. The survival time of the animals was less than an hour as shown by the curve.

The rate of survival of the animals injected with ADP treated with and without SNP “pad” were subjected to Kaplan-Meir survival test for determining the significance of the survival rate in these animals (Fig. 4). A comparison of the survival curves as determined by the Log-rank (Mantel-Cox) Test suggested that the survival curves were significantly different with a P value of 0. 0331. Additionally, Gehan-Breslow-Wilcoxon Test also determined the survival curves to be significantly different with a P value of 0. 0399.

### The effect of increased plasma NO level by using SNP “pad” on the rate of death due to AMI in subjects with different cancers

As the use of the SNP “pad” in the animals was found to protect the animals from death due to ADP induced thrombosis, the rate of death due to AMI was determined in cancer patients who had received the SNP “pad” at least for 3 years everyday for different kinds of cancers as described before [28]. It was reported that while the plasma NO level was ≈4 µM in normal volunteers, the plasma level of NO in all cancers tested was found to be reduced to 0 µM [28]. In a total of 8,283 subjects with different kinds of cancers (Table 2) who received the dermal SNP “pad”, for at least 3 years the plasma NO levels was restored to ≈4 µM, (p<0.0001) as reported before [28]. Interestingly, the actual numbers of deaths due to AMI itself in these patients were only 895. As described in the Table 3, it was found that the death rate in normal population due to AMI was 40%. However the rate of death due to AMI in the cancer patients who had received the “pad” for 3 years was 12.265% that contrasted the death rate in the cancer patients (29.858%) who did not receive any SNP “pad”. Z-test analysis between the groups was performed where the calculated z-value of the death rate due to AMI between the normal population and the cancer patients using SNP “pad” was 17.239 which was greater than the critical two-tailed z-value 1.159. Also, the z-value of the death rates due to AMI between the cancer patients not receiving the SNP “pad” and the cancer patients using the “pad” was 22.3 which was again greater than the critical two-tailed z-value 1.959. The P-value for the two-tailed test in all the cases was found to be 0 which was less than the alpha value of 0.5 (i.e., P<0.5). Z-test analysis therefore established that the rate of death due to AMI in the participating cancer patients was significantly lower even than that in the normal population that contrasted a significant increase of death rate due to AMI in cancer patients.

**Table:** **Table-2.** Cancer entities of the patients selected for the study.

Types of cancer	Age (years)	Gender and number of patients who participated in the study
Lungs non-small cell carcinoma	45–72	Male (135); female (75)
Lungs small cell carcinoma	30–65	Male (75); female (70)
Breast cancer	25–55	Female (1050)
Esophagus	35–50	Male (450); female (159)
Stomach	28–60	Male (255); female (101)
Liver	28–60	Male (150); female (100)
Pancreas	28–65	Male (300); female (175)
Gall bladder	30–65	Male (110); female (150)
Colon	35–71	Male (175); female (92)
Rectum	34–68	Male (165); female (97)
Acute lymphoblastic leukemia	4–70	Male (105); female (150)
Acute myeloid leukemia	5–75	Male (55); female (45)
Multiple myeloma	30–60	Male (65); female (15)
Non-Hodgkin's lymphoma	24–58	Male (65); female (110)
Hodgkin's Lymphoma	17–52	Male (85); female (100)
Uterus	35–60	Female (1020)
Cervix	35–60	Female (998)
Renal cell carcinoma	35–70	Male (65); female (80)
Ovary	25–60	Female (1091)
Prostate	35–75	Male (145)
Glioma	30–72	Male (115); female (90)

The study involved the application of SNP “pad” for 3 years in patients suffering from different kinds of cancer. The digits within the parentheses denote the number of patients who volunteered to participate in the present study.

**Table 3:** Death rates due to acute myocardial infarction in cancer patients with and without the use of SNP “patch” and in the normal counterparts.

Patients	Death rate due to occurrence of AMI	Significance using Z-test analysis
aNormal Subjects	40%	z-value between a and b = 17.2 (P<0.5)
bCancer patients using SNP “patch”	12.265%	z-value between a and c = 31.80 (P<0.5);
cCancer patients who did not receive SNP “patch”	29.858%	z-value between b and c = 22.3 (P<0.5);

a Data cited in Harrison's Principles of Internal Medicine, 16^th^ Edition, Volume 2, Chapter 208, Approach to the Patient with Cardiovascular Disease by Eugene Braunwald pp 1301, McGraw-Hill Medical Publishing Division.

b Data cited in *Sinha AK*, et al. Neutralization by “*antineoplastin*” of insulin-activated nitric oxide synthase antibody and its effects in cancers. J Cancer Res Clin Oncol. 2002 Dec;128(12):659–68.

c Data cited in Harrison's Principles of Internal Medicine, 16^th^ Edition, Volume 1, Chapter 66, Approach to the Patient with Cancer by Dan L. Longo pp-435. McGraw-Hill Medical Publishing Division and other journal articles reporting the statistical significance of the occurrence of AMI in cancer patients.

Sodium Nitroprusside “pad” was prepared and used dermally in patients with different kinds of cancers (n = 8,283) as described in the Materials and Methods in details.

Death rates due to AMI in the cancer patients who received SNP “pad” for 3 years was compared to the rate of death due to AMI in the normal population as reported in the literature.

The death rates between the groups were compared by using Z-test, a special case of null hypothesis.

## Discussion

These results suggested that a severe reduction of the plasma NO level could result in the worst prognostic outcome of AMI, in that, NO was not only a potent inhibitor of platelet aggregation [10] and an *in situ* thrombolytic agent [20], [25], but the abnormal reduction of the plasma NO level could also play a critically important role in the development of severe chest pain that is usually associated with AMI for which no acceptable mechanism is currently available. As NO generating compounds like organic “Nitro” compounds including isosorbate dinitrate and nitoglycerine *in vivo* have significant anti-anginal effect through the systemic generation of NO in the victims of AMI [39], it could be inferred, as a corollary, that the severe reduction of the plasma NO level to 0 nmol/ml, might have a significant and a contributory role in the development of the cardiac pain associated with AMI. In this context, it could also be mentioned that while the male gender hormone, testosterone, is an inhibitor of systemic NO synthesis, estrogen, the female gender hormone in contrast, was a potent activator of nitric oxide synthase (unpublished). The effect of estrogen on the stimulation of NO in female might offer an explanation for the usual lack of severe chest pain in females due to the precipitation of AMI that sharply contrasted the high intensity pain that occurs in the case of male AMI victims. We have also reported that the infusion of insulin in the AMI victims favourably reduced the anginal pain including unstable angina [40].

The severe reduction in the plasma NO level in AMI cannot possibly be related either to the ethnic background of the subjects or to the geographical location of the patients. The reduction of the plasma NO level was essentially similar both in the cases of the patients from northern India and in southern Australia (Fig. 2A, 2B).

Furthermore, the increase of NO would not only inhibit platelet aggregation [10] but might work as a thrombolytic agent through the direct activation of plasminogen to plasmin in the absence of any factor or cell independent of Hageman factor dependent synthesis of the enzyme in the intrinsic pathway of blood coagulation [20]. The increased NO synthesis has also been reported to stimulate insulin synthesis in the liver even when the pancreatic β cells were non-functioning [27]. The increase of insulin synthesis which itself is a potent antithrombotic humoral factor [11], has been reported to result in better prognostic outcome due to the control of hyperglycemia which is reported to be associated with the increase in the infarct size in AMI [41]. As presented in the Results, it was found that the appearance of dermcidin isoform 2 (dermcidin) in the plasma of AMI patients was found to be always associated with the condition [36]. As mentioned above, dermcidin was a competitive inhibitor of all known forms of nitric oxide synthase (NOS) where *l*-arginine is the only substrate known, the appearance of dermcidin in the circulation of AMI would result in the systemic inhibition of NO synthesis, and, as such, dermcidin could be critically important in the reduction of plasma NO level in AMI [36]. Furthermore, we have also found that ADP itself was a potent inhibitor of nitric oxide synthase (unpublished), and, consequently, the platelet aggregating agent might also be responsible not only in the initiation of thrombus formation on the arterial wall in human AMI [2], but the compound might have an important contributory role in the reduction of the plasma NO level in AMI leading to the cardiac pain associated with the condition.

Although we could not use SNP “pad” as a protective agent against AMI in normal population, our results nevertheless indicated that the systemic increase of NO reduced the death rate in cancer patients who were actually reported to be at a significant greater risk of developing AMI compared to general population [29]–[33]. It has been reported by numerous investigators that the co-existence of different kinds of cancer predispose the patients to the increased occurrences of death due to AMI compared to normal population [29]–[33]. The death rate among all different kinds of cancers victims is not available. However the death rate in some of the cancers for e.g., the death rate in Hodgkin's lymphoma [29], breast cancer [30], prostrate cancer [33] and other forms of cancer was markedly higher due to AMI compared to that in the normal population. Several studies have shown the efficacy of various thrombolytic drugs like aspirin and warfarin as well as dalteparin in different acute thrombotic events in patients with myeloma and pancreatic cancer although the mechanisms of action of these drugs remain yet to be determined [42]–[43]. It should be mentioned that, it was not possible for us to determine the effect of SNP “pad” in the prevention of death due to AMI in all known kinds of cancers compared to normal population. However, the beneficial effect of our invention could be achieved by patients suffering from various kinds of cancers (Table 3) at a nominal cost following the basic rule of GMP. As such, the use of SNP “pad” might be useful to protect persons from death particularly who are at high risk for developing AMI due to the reduction of the plasma NO (Fig. 2A; 2B).

In this context, it should be mentioned that although appropriate scanning procedures eg., ultrasonography or Computed Axial Tomography would be able to demonstrate the presence of atherosclerotic plaque in the arteries of the heart, unfortunately it is not yet possible to predict whether or when, if any, of the atherosclerotic plaques might rupture to precipitate the AMI. It should be mentioned here that even the mechanism of plaque rupture itself remains obscure. Indeed persons who are considered to be at high risk for developing AMI, as in the cases of diabetes mellitus and hypertension or have a family history for the occurrence of AMI, the use of aspirin is reported to reduce the incidences of occurrence of the condition [21], [22]. However, long term use of aspirin (sometimes within months) may result in tachyphylaxis (resistance) to the compound. It should also be mentioned here that it is not only the inhibition of cyclooxygenase through which aspirin exerts its effect, but as we have reported before, aspirin was a potent stimulator of NO synthesis in platelets [25]. In that sense, SNP “pad” might be a better alternative as an inhibitior of platelet aggregation as well as a thrombolytic agent since it does not produce tachyphylaxis even after the continuous use of the “pad” for 3 years. As NO is a physiologic compound and is involved extensively in various metabolic activities of the system, unlike aspirin (a pharmacologic agent), NO does not produce tachyphylaxis as physiologic agents very seldom are known to cause tachyphylaxis. Our experience indicated that SNP “pad” did not result in the development of resistance to the NO effects. In any event SNP “pad” might be an alternative to the use of aspirin, particularly in those cases where the use of aspirin could be a contraindication. Our results suggested that the use of SNP “pad” in these cases might be beneficial even when used for years for the prevention of the impending precipitation of AMI due to atherosclerotic plaque rupture/fissuring as an alternative to aspirin.

Finally, the efficacy of the SNP “pad” for the systemic increase of the plasma NO level was due to the unique “breeder” enzymic property of the insulin activated nitric oxide synthase (IANOS) [34] and not due to the administration of any pharmacologic agent in the system to increase NO levels. The NO induced production of NO catalyzed by IANOS was a self controlled process, in that, the increase of NO beyond 4.0 µM resulted in the inhibition of further NO synthesis [28]. In other words, there would be no “over production” of NO synthesis due to the “over” use of SNP “pad”.

## Conclusion

The systemic decrease of plasma NO level could be an important factor in the development of AMI. In consequence, when the impaired NO level was restored to normal ranges (0 µM v/s 4.0 µM) in cancer patients, it resulted in the decreased incidences of AMI compared to normal population as indicated by statistical analysis. It is therefore possible that the dermal application of SNP “pad” might be useful in reducing the death rate in normal population in general.

## Supporting Information

Checklist S1 **Trend Statement Checklist.** A total of 8,283 patients suffering from different kinds of cancers participated in the study. The procedure involved in the individualization of each patient and the administration of 0.28 mmol SNP/“pad” to these patients have been described in the text in details. The statistical significance was determined by a professional statistician and calculated by Student's Tau (**τ**) Test by using “null hypothesis”.
(PDF)

Protocol S1 **Details of the protocol of the study as approved by the Institutional Review Board for Human Research, Sinha Institute of Medical Science and Technology, Kolkata, India.** The protocol as approved by the IRB for Human Research, Sinha Institute of Medical Science and Technology, Kolkata, is self explanatory. The methodology followed for the use of “antineoplastin” pad in patients with different kinds of cancers is discussed elaborately in Protocol S1. Appropriate permission was obtained from the Calcutta High Court prior to the commencement of the trial. Each patient was individualized and they could terminate their participation from the study without prior approval from the investigators.
(PDF)
