# Supplementary material for: Reduction of Death Rate Due to Acute Myocardial Infarction in Subjects with Cancers through Systemic Restoration of Impaired Nitric Oxide
Source: PLoS One. 2014 Feb 18;9(2):e88639. doi: 10.1371/journal.pone.0088639 (PMC3928291; doi:10.1371/journal.pone.0088639)
Supplement: Protocol S1 — Details of the protocol of the study as approved by the Institutional Review Board for Human Research, Sinha Institute of Medical Science and Technology, Kolkata, India. The protocol as approved by the IRB for Human Research, Sinha Institute of Medical Science and Technology, Kolkata, is self explanatory. The methodology followed for the use of “antineoplastin” pad in patients with different kinds of cancers is discussed elaborately in Protocol S1. Appropriate permission was obtained from the Calcutta High Court prior to the commencement of the trial. Each patient was individualized and they could terminate their participation from the study without prior approval from the investigators. (PDF) [file pone.0088639.s002.pdf]

INSTITUTIONAL REVIEW BOARD (HUMAN & ANIMAL RESEARCH ETHICS COMMITTEE)
SINHA INSTITUTE OF MEDICAL SCIENCE AND TECHNOLOGY

Recognized Institute of Medical Science Research
By the Ministry of Science & Technology, Govt. Of India, New Delhi
288 Kendua Main Road, Baisnabghata, Patuli, Kolkata-700084
Ph.:+91-9903792100

Antineoplastin/cancer/clinicaltrial, No. A.S.T. 737 of 1999/S

Date: 15th July, 1999

To,
Prof. Asru K. Sinha, D.Sc.
Director
Sinha Institute of Medical Science and Technology
288 Kendua Main Road, Baisnabghata, Patuli, Kolkata-700084

Subject: Approval for the use of antineoplastin in human beings with different types of cancers (Antineoplastin/cancer/clinicaltrial, No. A.S.T. 737 of 1999/S)

Dear Prof. Sinha,

As the leading member of the investigation committee involving the study of your discovery of antineoplastin as a research material, each member approved that:

1. Antineoplastin is not a "drug" but an anti-cancer research material. And as such, you may use this research material only as a dermal patch on the skin of the volunteers with cancers only. This antineoplastin cannot be used by any other means including oral, or through injecting the material in the system and you must not use any anti-cancer drugs along with your antineoplastin therapy without our prior approval.
2. The volunteers who will participate in the study must be adults over the age 18. No mentally retarded, prisoner or pregnant women may participate without our consent. All volunteers participating in the study must obtain a magisterial affidavit (judicial), but not notarized affidavit, in the court of law being witnessed by a family member and advised by

INSTITUTIONAL REVIEW BOARD
SINHA INSTITUTE OF MEDICAL SCIENCE & TECHNOLOGY
ESTD - 1995
GARIA, KOLKATA - 700 084

a legal counselor regarding the appropriateness of the person to be a volunteer in the study. A copy of the judicial affidavit that you must follow is attached herewith for your convenience. Under any condition/s, you must not stay away from these conditions as described above.

3. If the participant is already under some care of a physician, you must obtain the consent of the attending physician before you obtain judicial permission from the court of law. All the participants should be routinely asked to get the reports of their complete blood picture and liver function test.
4. Participants will not be compensated for participation in the study and you are also not allowed to impose any charge for delivering your antineoplastin. You must follow GMP as stipulated by the Indian Medical Association and American Medical Association, i.e., you are not permitted to ask for excessive price that cannot be legally approved for public consumption.
5. Should any patient/participant get unwell due to their involvement in the study, you will have the legal/medical obligation to take care of the person.
6. Please note that, we have approved the use of this anti-cancer research material (sodium nitroprusside) in human beings because it has already been approved by the FDA to be used for malignant hypertension. The FDA law stipulates that any medicine already in use to treat a condition can be used in any other condition without further approval from the FDA. This approval is based on the safety of the therapy and not the efficacy of the treatment.
7. The committee has the right to inspect the progress and problems of your investigation involving the use of this research material which can be revoked, if necessary, within 24h.
8. All the experiments involving animals must follow safety and care with human kindness. All the animals must be housed in well-lighted and clean animal facility and treated humanely to minimize pain. If required, an activist of the Animal Rights Group may be present during the experiments to monitor the treatment of the animals. The animals must be routinely checked by a certified veterinarian to be free from any disease for their use in the study. The termination of the study strictly requires that the animals must be sacrificed with minimum pain as possible preferably by euthanasia using a CO_2 chamber.

Approved by the members of IRB, SIMST:

1) (Honorary Chairman of IRB) Udayan Ray 19/07/99
Dr. Udayan Ray MD. PhD., Royal Hobart Hospital, University of Tasmania, Australia

INSTITUTIONAL REVIEW BOARD
SINHA INSTITUTE OF MEDICAL
SCIENCE & TECHNOLOGY
URoy ESTD - 1995
GARIA, KOLKATA - 700 084

2) Anil Pattanayak L.L.B (Calcutta High Court) Anil Pattanayak 15.07.99

3) Mrs. Madhuri Ghosh, L.L.B (Calcutta High Court) Madhuri Ghosh 15.7.99

4) Mr. Soumen Maiti L.L.B (Calcutta High Court) Soumen Maiti 15.07.99

5) Mr. Chttaranjan Kundu (Newspaper Editor) CKundu 15.7.99

6) Dr. Gopal Majumder, PhD. Scientist-G, Indian Institute of Chemical Biology, Kolkata, India. G Majumdar 15.7.99

7) Prof. Rabindra Bhattacharya, DNB Med; DM CARD; DNB CARD, Prof & Head, Dept of Cardiology, Calcutta Medical College, Kolkata, India. RBhattacharya 15.7.99

8) Dr. Gorachand Bhattacharya M.D., Prof & Head, Dept of Biochemistry, Calcutta National Medical College, Kolkata, India. Gorachand Bhattacharya 15.7.99

9) Dr. Sahana Majumder PhD. Assistant Professor, Dept of Physiology, Rammohan College, University of Calcutta Sahana Mazumder 15/07/99

10) Dr. Gausal A. Khan, PhD. Scientist, Dept of Physiology, DIPAS, Delhi, India Gausal A. Khan 15/7/99

11) Mr. Rudraj Ghosh, Businessman, Shillong, Meghalaya, India. Rudraj Ghosh 15/7/99

INSTITUTIONAL REVIEW BOARD
SINHA INSTITUTE OF MEDICAL
SCIENCE & TECHNOLOGY
ESTD - 1995
GARIA, KOLKATA - 700 084
